# Extrahepatic Manifestations of Chronic HBV Infection and the Role of Antiviral Therapy

**DOI:** 10.3390/jcm11216247

**Published:** 2022-10-23

**Authors:** Cesare Mazzaro, Luigi Elio Adinolfi, Gabriele Pozzato, Riccardo Nevola, Ada Zanier, Diego Serraino, Pietro Andreone, Roberta Fenoglio, Savino Sciascia, Valter Gattei, Dario Roccatello

**Affiliations:** 1 Clinical of Experimental Onco-Haematology Unit, Centro di Riferimento Oncologico di Aviano (CRO) IRCCS, 33081 Aviano, Italy; 2 Unit Internal Medicine, Department of Advanced Medical and Surgery Sciences, Luigi Vanvitelli University of Campania, 80138 Naples, Italy; 3 Department of Clinical and Surgical Sciences, Maggiore Hospital University of Trieste, 34149 Trieste, Italy; 4 Department of Internal Medicine, Pordenone General Hospital, 33170 Pordenone, Italy; 5 Cancer Epidemiology Unit, Centro di Riferimento Oncologico di Aviano (CRO) IRCCS, 33081 Aviano, Italy; 6 Division of Internal Medicine, Department of Medical and Surgical Sciences, Maternal-Infantile and Adult, University of Modena and Reggio Emilia, 41124 Modena, Italy; 7 University Center of Excellence on Nephrologic, Rheumatologic and Rare Diseases (ERK-Net, ERN-Reconnect and RITA-ERN Member) CMID-Nephrology and Dialysis Unit, San Giovanni Bosco Hub Hospital, Department of Clinical and Biological Sciences, University of Turin, 10154 Turin, Italy

**Keywords:** hepatitis B virus, HBV extra-hepatic manifestations, HBV-related glomerulonephritis, HBV-related cryoglobulinemia, HBV-related vasculitis, entecavir, tenofovir

## Abstract

The hepatitis B virus (HBV) infection leads to chronic hepatitis, cirrhosis, and hepatocarcinoma. However, about 20% of patients experience extrahepatic manifestations such as polyarteritis nodosa, non-rheumatoid arthritis, non-Hodgkin lymphoma, cryoglobulinemic vasculitis, and glomerulonephritis. These influence the patient’s morbidity, quality of life and mortality. The treatment of an HBV infection is based on nucleotide analogues (NAs) which are safe and effective for the suppression of HBV-DNA in almost 100% of cases. A few studies have shown that NAs induce a viral response and an improvement of extrahepatic diseases. There is a lack of a thorough analysis of the available treatments for extrahepatic HBV manifestations. In 90% to 100% of cases, the NAs stop the HBV replication, and they produce a clinical response in the majority of patients with mild to moderate extrahepatic signs/symptoms. Arthritis can definitely disappear after the HBV elimination and, in some cases, the HBV eradication following NAs therapy appears to improve the renal function in HBV-related nephropathies. Plasma exchange can be used in subjects who are suffering from the most aggressive forms of cryoglobulinemic vasculitis and glomerulonephritis, progressive peripheral neuropathy, and life-threatening cases, and this can be combined with glucocorticosteroids and antiviral agents. In selected refractory patients, the use of rituximab in conjunction with NAs therapy can be considered. The review provides an update on extrahepatic conditions that are linked to HBV and the impact of treating HBV with NAs.

## 1. Introduction

Chronic hepatitis B virus infections affect about 250 million people globally. It is a hepatotropic virus, which in most cases evolves into cirrhosis and hepatocellular carcinoma. An annual rate of 887,000 liver-related deaths has been estimated [1]. A coincidental connection between a chronic HBV infection and several extrahepatic symptoms is being supported by increasing evidence, including cryoglobulinemic vasculitis, glomerulonephritis, serum sickness-like syndrome, polyarthritis nodosa, non-rheumatoid arthritis, and non-Hodgkin lymphoma (NHL) (Table 1) [2]. Extrahepatic symptoms may affect individuals’ morbidity, quality of life, and mortality. Nucleotide analogues (NAs), which are adopted to treat HBV infections, were proved to be safe and effective for HBV-DNA suppression in approximately 100% of the cases [3]. The NAs have been used to treat almost all of the chronic HBV infections, including cirrhosis, with a good efficacy and safety profile [3]. Several studies have shown a similar antiviral efficacy in some manifestations of HBV-related mixed cryoglobulinemia and a link between the treatment-induced HBV viral response and the improvement of other extrahepatic diseases, including arthritis and nephropathy [4] (Table 2).

This article provides an update on the extrahepatic conditions that are caused by HBV, and it discusses how NAs treatment can change the patient outcomes by eliminating HBV.

### 1.1. HBV-Related Cryoglobulinemia Vasculitis

The presence in the serum of cryoglobulins, which are immunoglobulins that precipitate when the temperature falls below 37 °C and redissolve when they are heated, is known as cryoglobulinemia. There are three different categories for cryoglobulinemias [10]. Multiple myeloma, Waldenstrom’s disease, and NHL are the examples of the lymphoproliferative conditions that are related with type I, which consists of a single monoclonal immunoglobulin, either IgM or IgG. In the type II and III, the cryoglobulins are immunocomplexes that are composed of polyclonal IgG and monoclonal or polyclonal IgM that are provided with rheumatoid factor activity. These forms are usually referred as mixed cryoglobulinemia (MC).

MC is a systemic form of vasculitis involving the small (and rarely medium)-size vasculature that is caused by the precipitation of circulating cryoglobulin [11]. MC occurs in the context of a HCV infection in nearly 90% of the cases [12], while 10% of them are secondary either to lymphoproliferative disorders or rheumatologic diseases, or HBV or HIV (or both) [4,13]. The symptoms of MC include purpura, asthenia, and arthralgia [14], but sensitive-motor peripheral neuropathy and glomerulonephritis can be present. The predominant renal manifestation of it has a typical membranoproliferative pattern. On occasion, MC evolves into low-grade NHL. In rare cases, the disease has a more severe, life-threatening presentation such as heart vasculitis, gastrointestinal vasculitis, or the involvement of the central nervous system [15].

Levo and colleagues [16] made the initial suggestions on the potential contribution of a chronic HBV infection as an MC etiologic agent more than 40 years ago. The frequency of chronic HBV infections in MC has now been documented in various series over the past 20 years, with estimates ranging from 0.5 to 5.5% [17,18,19]. The incidence of CV in the HBV participants has not yet been examined in any study. The few published studies on HBV-related MC have shown that 50% of the patients had chronic hepatitis while 30% of them had cirrhosis [4]. Between 45% and the totality of the reported cases had mild–moderate clinical manifestations of it (arthralgia, palpable purpura on the leg, or asthenia). Arthralgia is typically characterized by non-deforming, bilateral, symmetrical joint discomfort that primarily affects the knees. In 10% to 30% of the cases, skin ulcers can develop. There have also been reports of Raynaud’s phenomenon and Sicca syndrome. The peripheral nervous system is involved in a non-negligible number of the cases, which increases to as high as 60% of the cases if electromyography is employed. The most prevalent condition is a distal peripheral neuropathy with sensory or sensory-motor involvement. The patients report pain and asymmetric paraesthesia, with it subsequently becoming symmetric. The motor neuropathy is not constant, with it mainly affecting the legs, and it usually manifesting a few years after the sensory neuropathy. Similar to the HCV-related MC, the prevalent renal manifestation is membranoproliferative glomerulonephritis. Low-grade indolent NHL is observed in 10% of the patients [9]. In rare cases, the disease is life-threatening, affecting the central nervous system, the gastrointestinal tract, and the heart [4].

### 1.2. Treatment of HBV-Related Cryoglobulinemic Vasculitis

**Figure 1:**
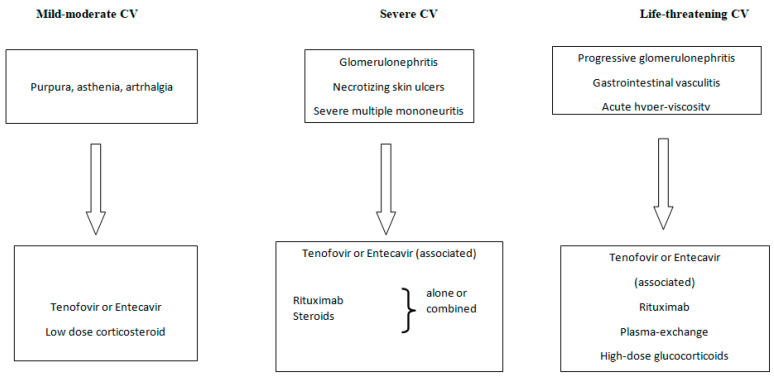
Flow-chart of HBV- related cryoglobulinemia vasculitis therapeutic management.

There are no specific recommendations for the treatment of HBV-related cryoglobulinemic vasculitis that are available. However, similarly to HCV-related CV, the following approaches can be considered (Figure 1):

1. Antiviral therapy (oral nucleot(s)ide analogues);
2. B-cell depleting therapy (Rituximab);
3. Glucocorticosteroids;
4. The removal of circulating cryoglobulins by plasma exchange;
5. Nonsteroids anti-inflammatory agents.

The first line of treatment for an HBV chronic infection is its eradication or the successful suppression of it by NAs.

The significant highlights on the use of NAs in the management of HBV-related CV are summarized in Table 1.

Compared to the virologic response, the clinical and immunological responses are noticeably less remarkable. In a consistent number of cases, after receiving the NAs therapy, the vasculitis symptoms such as minor purpura, asthenia, and arthralgias may vanish or improve [7,9]. The NAs can eradicate the virus in patients with sensory motor peripheral neuropathy, but they frequently remain as clinical non-responders. Rituximab (RTX) can obtain a clinical and immunological improvement [9], but its use can be harmful.

In a few studies with HBV-related glomerulonephritis, some improvement of the renal function and urinary abnormalities have been reported following NAs therapy [6,9,20]. However, the majority of the patients eventually underwent RTX therapy or aphaeresis, and in a way that is similar to HCV-associated MC, they may achieve immunological and functional improvement [21,22]. As for the HCV-related cryoglobulinemic glomerulonephritis, RTX remains the first-line immunosuppressive agent. Similar to this, the low-grade NHL patients who received NAs required subsequent chemotherapy or immunotherapy since they did not experience a hematological response [9,23].

Pegylated interferon has an ancillary role in HBV-related CV [24].

In clinical practice, the vasculitis symptoms are frequently treated with the long-term administration of low-medium glucocorticoide dosages. Corticosteroids have been utilized in case reports of aggressive systemic CV (progressive peripheral neuropathy and glomerulonephritis) at high dosages or as a pulse therapy [6,9]. To treat a severe CV flare, high dosages of corticosteroids may be recommended, either alone or in combination with aphaeresis [25].

In a recent report, two patients who received entecavir and RTX together experienced a renal disease remission and an HBV-DNA suppression [6]. After receiving the tenofovir treatment for low-grade NHL, a patient underwent a subsequent treatment with plasma exchange and low-dose rituximab, which resulted in a partial response [9]. There are not many studies showing that rituximab is beneficial in treating peripheral neuropathy, non-responders to antiviral treatments, or relapsers [9].

Because there is a chance that HBV-positive individuals, including those who have had a prior infection (HBcAb-positive alone), could suffer a reactivation of it, antiviral therapy with NAs must always be combined with rituximab [3].

A severe and potentially fatal HBV-related CV may require plasmapheresis (such as those cases with extended skin ulcers, hyper-viscosity syndrome, progressive peripheral neuropathy, or progressive glomerulonephritis).

In conclusion, antiviral therapy with NAs alone should be limited to those cases with mild sign/symptoms that are associated with HBV-related CV. RTX should be considered as a first choice in glomerulonephritis, prevalent sensitive-motor peripheral neuropathy, and relapsing patients. RTX that is associated with plasmapheresis and high-dose corticosteroids can be considered in life-threatening cases.

## 2. HBV and Rheumatologic Manifestations

In both the acute and chronic phases of the disease, an HBV infection is linked to a broad range of rheumatologic symptoms. The mechanisms that are involved in these rheumatic manifestations seem to be primarily related to immune-mediated phenomena. The most frequently reported HBV-associated rheumatologic manifestations are serum sickness-like syndrome, arthritis, polyarteritis nodosa (PAN), and essential mixed cryoglobulinemia (discussed above). Furthermore, fibromyalgia and muscle pain have been described in about 3% of the cases, Raynaud’s phenomenon has been described in 2% of them, and Sjogren’s syndrome has been described in 3% of them [26]. Additionally, the positivity for rheumatoid factor (RF) or other autoantibodies is found in a number of cases.

### 2.1. Serum Sickness-like Syndrome

It has been reported as an extrahepatic manifestation that is associated with an acute HBV infection in up to 30% of the patients during the prodromal phase of the condition. The rheumatologic manifestations are characterized by “non-destructive” arthritis and arthralgia. Feet and hands, in particular the small joints of them, are usually symmetrically involved, with swelling, edema, and pain as the main features. Although cases involving the large joints, such as the knees and ankles, and with asymmetrical localizations of it have also been described. The rheumatologic symptoms are associated with generalized malaise, fatigue, fever, muscle pain, and erythematosus skin rashes such as palpable purpura, maculopapular lesions, and multiform erythem [27]. The pathogenesis appears to be due to circulating immune complexes containing HBsAg [28]. The syndrome precedes the onset of the symptoms of an acute HBV infection, and it can last from a few days to months, with it tending to regress with the onset of the symptoms of hepatitis. In some cases, arthritis can persist or occur periodically and its definitive disappearance occurs only with the elimination of HBV.

### 2.2. Non-Rheumatoid Arthritis

Non-rheumatoid arthritis is a frequently reported manifestation during an HBV infection, and it is typically asymmetric, and it may be associated with skin erythema, and usually, this appears within the first 3 months of the hepatitis onset, but occasionally, it can be observed also in the chronic phase of the infection [29]. Its pathogenesis is characterized by the synovial deposition of circulating immune complexes containing HBsAg-HBsAb and an activated complement [30]. The symptoms can appear simultaneously in all of the affected joints or have the characteristics of being migratory or additive. In a high percentage of the HBV cases, there is monoarticular involvement affecting the large joints, while in a smaller percentage, it affects the small joints, although combined cases have been be observed. The presence of anti-CCP is detected in a small percentage of the cases (5%), ANA is detected in 10% of them, and the presence of RF is detected in about a quarter of the cases. Non-steroidal anti-inflammatories are typically effective in treating this arthritis within a few days. In 4% of the cases, true rheumatoid arthritis is related to HBV infection [31].

### 2.3. Polyarteritis Nodosa (PAN)

HBV-associated PAN may occur at all of the stages of an HBsAg infection. It is present in 1–5% of the cases of HBV infections, while 35–50% of the patients with PAN are positive for HBsAg [32,33]. Notably, the prevalence of HBV-related PAN appears to have fallen to less than 5% with the introduction of the HBV vaccine [34]. The precipitation of circulating immune complexes containing an excess of HBV antigens seems to be one of the pathogenic mechanisms underlying PAN [35]. The disease activity has been found to be related with the circulating immune complex levels [36]. HBV-associated PAN usually is observed at the beginning of the infection [37], and clinically, it is no different from the idiopathic form, and it includes polyarthritis, polyarthralgia, a rash, a fever, livedo reticularis, abdominal pain and diarrhea, weight loss, peripheral nervous system involvement, hypertension, renal failure, and coronary vessel involvement. However, when it is compared to the non-HBV-associated form, it is characterized by a more pronounced involvement of the gastrointestinal tract (often associated with hemorrhage and perforation), increased testicular involvement, malignant hypertension, polyneuropathy, and heart disease. Anti-neutrophil cytoplasmic antibodies (ANCAs) should be absent by definition. The levels of the complement components are usually normal. In these patients, the hepatitis activity is generally mild at the time of the onset of the PAN symptoms, and serum transaminase is reported to be within range in about half of the patients [37]. HBV-related PAN is characterized by fewer relapses, but it has a higher mortality rate than the non-HBV form [33].

In regard to the renal involvement, it is characterized by hypertension and functional impairment. Urinary abnormalities are ancillary, and they more often consist of isolated mild proteinuria. In this form of vasculitis, the glomerular loops appear to be hypoperfused when they are examined using the scanning microscope, but they do not present with signs of inflammation. The pathogenesis of it is due to the phlogistic sequelae of the deposition of the immune reactants, thereby resulting in the collapse of the vessel wall and the formation of microaneurysms. By definition, small vessels, including the glomerular loops, are spared from the inflammation. The decrease in glomerular filtration is a consequence of the hypoperfusion; the diagnosis of this is clinical, wherein the inflammation biomarkers are elevated, leukocytosis is common, and on occasion, the circulating eosinophils can be increased. Angiographic examinations of the involved organs are of upmost importance. Ultrasound techniques can be the initial approach, but an angio-CT scan, angio-magnetic resonance, and especially, arteriography are essential. Due to size of the affected vessels, positron electron tomography can be less efficient than when it is used for the large vessels. The hepatic, pancreatic, splenic, mesenteric, and renal arteries are the more commonly affected ones. However, the coronary arteries can be affected too. The diagnostic hallmark of the disease is the detection of multiple aneurisms in the affected organs. Ischemic or hemorrhagic sequelaes with infarction or hematomas are common [34].

Combinations of antiviral drugs, glucocorticosteroids, and plasmaexchange have been suggested for the management of HBV-associated PAN [38,39].

### 2.4. Laboratory Rheumatologic Abnormalities

In regards to the laboratory abnormalities, a high prevalence of RF (approximately 12%) has been observed in the HBsAg-positive subjects [40], and its titer correlates with the serum-HBV DNA levels [40]. Autoantibodies are frequently detected in the serum of the HBsAg-positive subjects independently of the liver disease’s phases. ANAs were reported in about a quarter of the subjects, and AMAs, anti-SMA, anti-LC-1 and anti-LKM-1 were reported in 7%, 3%, 3%, and 1% of them, respectively, while anti-Ro52 was reported in about 30% of them, anti-gp210 and anti-PMl were reported in 10% of them, and anti-SLA/P and anti-Sp100 were reported in about 3% of them [41].

Last, but not least, it should be noted that the anti-HBV vaccination can cause autoimmune reactions that are characterized by arthritis, rheumatoid arthritis, and SLE [42].

## 3. Renal Manifestations of Hepatitis B Virus Infection

In general, 3–5% of the people with a persistent HBV infection may develop renal manifestations. Acute GN is a direct pathogenic relationship between active viral replication and viral-associated glomerulonephritis, according to its definition [43,44].

Children and men are more likely to develop glomerular disorders. Membranous nephropathy and membranoproliferative glomerulonephritis are the two types of glomerulonephritis that are most frequently seen. Additionally, the documented conditions include focal segmental glomerulosclerosis and IgA nephropathy. PAN, extra capillary GN, and minimum change disease have also been documented [45]. The presence of glomerular immune deposits containing one or more HBV antigens that are detectable using immunohistochemical methods, a persistent HBV infection, and serologic evidence are all required to make the diagnosis of HBV-related GN. Immune complex deposition in the glomeruli is the pathogenetic process that is most frequently acknowledged. It is debatable, however, whether the immunological complexes develop in situ or come from the circulation of blood and become locked in the glomerular architecture [46]. It is important to note that many people with chronic HBV may also have coexisting HIV (5–10%) and HCV (10–30%), which could influence the glomerular pathology that manifests and further widen the clinical differential diagnosis. During the evaluation process, it is crucial to check for these other latent viruses in all of the HBV carriers [47].

### 3.1. HBV-Related Membranous Nephropathy (MN)

Children and adults have varied natural histories of membranous nephropathy which are driven by HCV. Children frequently experience spontaneous proteinuria remission and the long-term preservation of the renal function. Up to 30% of the patients may develop renal failure, and adults are more predisposed to the disease progressing. C3 and C4 are reduced in up to half of the cases. Histologically, HBV-related MN is characterized by a thickness of the walls of the capillary loop, which in the advanced cases, can take on a stiffened appearance [48]. The presence of circulating anti-phospholipase A2 receptor antibodies (anti-PLA2R) helps to distinguish the idiopathic MN from the HBV-associated MN. Additionally, the identification in the glomerular basement membrane of the IgG subtype can be informative: in the HBV-associated MN, it seems to be made up of, predominantly, the IgG1 class, while in anti-PLA2R MN, it tends to be the IgG4 class [49].

**Figure 2:**
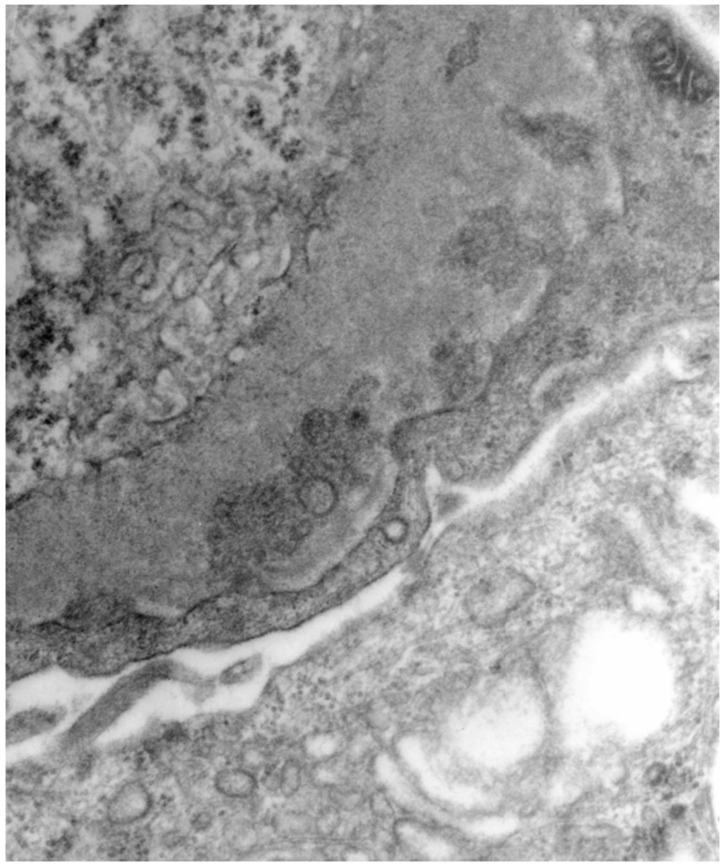
Microsferular substructures (so called virus-like particles) can be appreciated inside the electron-dense deposits of the subepithelial side of the glomerular basement membrane.

C3, IgG, and less often IgM in the granular deposits which can be observed in the subepithelial space are usually detected by immunofluorescence. When they are compared to the idiopathic forms, the deposits in the HBV-related ones can also be observed at the mesangial level. Extensive foot processes effacement can be observed when one is using electron microscopy. Viral particles (Figure 2) can be detected in different glomerular sites [50].

### 3.2. HBV-Related Membranoproliferative Glomerulonephritis (MPGN)

MPGN is frequent in HBV carriers. It does not present characteristics of specificity. A mesangial expansion, a thickness of the capillary wall and its double contours, a mesangial interposition, and endocapillary hypercellularity with the recruitment of monocytes and neutrophils are the main features. Immune deposits consisting of IgG, complement components, and IgM are detected in the subendothelial, mesangial, and paramesangial spaces by immunofluorescence [51]. Membranoproliferative glomerulonephritis is also the main histologic presentation of the HBV-related cryoglobulinemic renal involvement (see above). Electon microscopy in these rare cases shows the structured deposits.

### 3.3. General Principles of Therapy of HBV-Related Nephropathies

Most of the insights on the frequency of the response to regimens to manage HBV-related glomerulopathies derive from the experiences in cohorts of patients with MN. Data on MPGN and other forms of renal involvement are related to small studies/case reports. The treatment is the virus’ eradication. Lamivudine has been proven to have some effectiveness in reducing proteinuria, especially in children who experience active viral replication, elevated levels of HBV-DNA, and active hepatitis.

Glucocorticosteroids are used as a rescue therapy in combination with anti-viral agents. Immunosuppressive treatments are contraindicated, unless they are used in combination with antiviral agents [51,52,53,54,55,56]. A special warning has been reserved for Rituximab regimens. In a limited number of cases and in the absence of HBV-DNA, it might be administered in association with entecavir. However, RTX has a special role in the rare cases of HBV-related cryoglobulinemic glomerulonephritis. High doses of immunoglobulins (0.4 g/day of intravenous IgG in different regimens, i.e., either single cycle of five days or 3-monthly cycles of three days, followed by single monthly infusions for 9 months) have been anecdotally used in membranous nephropathy and PAN with renal involvement.

## 4. HBV and Lymphoma

Hepatocellular carcinoma and acute and chronic hepatitis can both be caused by the hepatitis B virus. Geographical differences have a substantial impact on the prevalence of HBV infections. According to the estimates, its prevalence is roughly 1% in Western nations and it is over 2% in the majority of the developing nations, with there being peaks of over 8% in some Asian and African nations [57]. Less research has been performed on the relationship between NHL and HBV than that which has been performed on HCV. In the same year as the initial report on the association between HCV and NHL, a report on an association between HBV and NHL was also published [58].

Numerous case–control studies involving a non-negligible patient population have been reported [59]. According to the findings of a study that was performed in Korea, 12.6% of the patients with NHL had positive HBsAg levels, which was compared to 7.3% of those with non-hematologic malignancies [59]. The population in an Italian case–control research was the same as that which was examined at for HCV infection. The study included 392 people in the control group and 400 B-NHL cases. The findings showed that the prevalence of HBV infections was considerably greater in the B-NHL cases than it was in the control group. Of the patients with B-NHL of HBsAg was represented in 8.5% compared to the 2.8% found in controls (adjusted odd ratio, OR: 3.67) [60]. Both the aggressive and indolent B-NHL showed the same positive association. In this study, 25.1% of the NHL patients had HCV. HCV/HBV co-infected four out of three hundred and ninety-nine B-NHL cases, and there were no controls in this study [60].

In cohort investigation of 603,585 South Korean people who were tested for HBsAg at a health screening, 8.8% of them were HBsAg positive at the baseline [61]. When they were followed over a period of 14 years, they observed that the risk of NHL elevated in the HBsAg-positive partecipants (HR: 1.74; 95% CI: 1.45–2.09). The risk reached a statistical significance only for the two most prevalent NHL subtypes (namely, diffuse large B-cell lymphoma (HR: 1.82, 95% CI: 1.34–2.48) and other/unknown non-Hodgkin lymphoma (HR: 1.49; 95% CI: 1.17–1.91)).

Being HBsAg positive was not associated with elevated risks of follicular and T NHL, Hodgkin’s lymphoma, and multiple myeloma.

If we include the patients with an occult hepatitis infection, the true link between HBV and NHL may be understated. The patients who tested negative for HBsAg but positive for HBV-DNA in their serum, tissues, or in both may have occult HBV infections [62]. According to several authors who investigated at the blood HBV-DNA in the patients with HBsAg-negative B cell NHL, the patients with NHL were at a considerably greater risk of occult HBV infection (6%), especially those with diffuse large B-cell lymphoma [63,64,65]. In a preliminary investigation [66] that was conducted in eight European nations, 739 incidents of NHL cases, 238 incidents of MM cases, 46 incidents of HL cases, and 2028 matched controls were included. Sero-positivity to HbsAg was not directly associated with the risk of NHL (OR = 1.78; 95% CI: 0.78–4.04) or HL (OR = 2.00; 95% CI: 0.13–32), but it was significantly associated with the risk of MM (OR = 4.00; 95% CI: 100–16.0) and the combination of three neoplastic conditions. Sero-positivity to anti-HCV was found to have non-significant direct associations with the risk of NHL (OR = 1.30; 95% CI: 0.55–3.07), MM (OR = 0.55; 95% CI: 0.6–5.39), and HL (OR = 4; 95% CI: 0.36–44.1). This study included controls from various nations with a HBsAg prevalence that ranged from 0% to 2.7% and anti-HCV infections that ranged from 0% to 5.3%.

The patients with an HBV infection did seem to have a higher probability of developing any form of cancer according to Andernsen and colleagues [67]. An HBV infection was linked to a higher risk of developing HCC (IRR = 9.6; 95% CI: 1.6–57.5), but not PC (IRR = 1.1; 95% CI: 0.1–9.2), NHL (IRR = 1.9; 95% CI: 0.4–9.0), or all of the other cancers (IRR = 1.2; 95% CI: 0.8–1.7). The target population in this investigation, however, displayed a relatively low prevalence of HBV chronic infections.

HbsAg was positive in 3.7% of the incident NHL cases and it was positive in 1.7% of the cancer-free matched controls in an Italian case–control research study [68], wherein the participants included 571 cancer-free incident NHL cases and 1004 cancer-free controls (QR = 1.95; 95% CI: 1.00–3.81). Being HBsAg positive was linked to a having a significantly higher risk of DLBCL, in particular (QR = 2.69), and all of the B-cell NHL subtypes (QR = 2.11, 95% CI: 1.30–5.59). The probability of follicular NHL was not substantially correlated with the HCV or HBV infections.

An updated meta-analysis of 58 papers was conducted by a Chinese study [69] to assess the relationship between HBV and NHL. The findings indicated that an HBV infection raised the risk of developing NHL by 2.50 times. The data from a stratified study show that B-cell NHL and HBV infections were more closely related to each other than T-cell NHL was to either of them. DLBCL and FL were strongly related with an HBV infection within the B-cell NHL subtype, but CLL/SLL and Burkitt lymphoma had no association with this.

The connection was stronger in the Asian and European nations with a medium–high HBV prevalence than it was in Oceania and the US with a low HBV prevalence.

The proportion of HBsAg-positive patients was significantly higher in the indolent version when it was compared with the aggressive B-NHL (59% vs. 38% *p* = 0.03) in a Chinese study of 72 patients with an HBV infection and B-NHL (aggressive 45 cases, 63%, and indolent B-NHL 27 cases, 37%). The individuals with indolent B-NHL had significantly higher HBV-DNA levels than the patients with aggressive B-NHL did (*p* = 0.01) [70].

In a large case–control research, Tian T [71] evaluated the link between HBV infections and several malignancies. Some of the patients (12.1%) and 5.5% of the controls were sero-positive for HBsAg. They reported strong associations between HbsAg sero-positivity and esofagus cancer (OR = 1.32; 95% CI: 1.13–1.54), stomach cancer (OR = 1.46; 95% CI: 1.30–1,65), HCC (OR = 39.11; 95% CI: 35.08–43.59), intraepatic bile duct carcinoma (ICC) (OR = 3.83; 95% CI: 2.58–5.67), The case–control study and meta-analysis that was conducted by these authors support the strong relationship between HBsAg sero-positivity and stomach cancer, ICC, ECC, PC, NHL, and leukemia.

One clinical investigation [72] examined the characteristics and prognoses of 39 individuals in low-incidence areas in France and Italy who had an active HBV infection with B-NHL. Twenty-four (62%) DLBCL and fifteen (38%) additional lymphoma subtypes mainly composed the distribution. Six patients had only a positive HBsAg result, and eight patients had only a detectable viral load among the 25 patients (65%) who tested positive for HBsAg. Before receiving the HNL diagnosis, none of the patients had a clinical history of cirrhosis. For 35 of the patients (90%), an antiviral medication was part of the treatment. Tenofovir (*n* = 8, 23%), lamivudine (*n* = 16, 46%), and entecavir (*n* = 11, 31%) were among the antiviral medications that were used. Following the antiviral medication use, HBV-DNA was suppressed in 19 patients (76%) out of the total. After an antiviral therapy that was used alone, they did not see any cases of a hematological response [72].

Cryoglobulinemia and other extrahepatic autoimmune conditions can be driven by both HCV and HBV. Both of the viruses are lymphotropic, and they can be found in mononuclear cells, and it has been investigated if they may directly cause cancer [73]. HBV, but not HCV, has been demonstrated to integrate into the host cell genome, thus causing a wide range of genetic changes [73].

The upturn of HCV-related NHL following the antiviral therapy has been largely documented, but so far, the antiviral therapy for HBV-associated NHL has never been evaluated [74].

Case reports have described the benefits of an antiviral treatment in patients who are affetced by a HBV infection that is associated with SMZL [75]. After the antiviral therapy with tenofovir disoproxil fumarate (245 mg), the suppression of HBV-DNA, the resolution of splenomegaly, and a bone marrow biopsy showed that there were no signs of lymphoma.

We recently reported one case of chronic hepatitis B-related low-grade B-cell NHL with aggressive features of cryoglobulinemic vasculitis (neuropathy, skin ulcers, and diffuse purpura), which was treated with tenofovir. The patient had no hematologic response and thus, they underwent a subsequent therapy with plasmapheresis and low-dose rituximab, which achieved a non-complete hematologic response and the rapid improvement of the vasculitis manifestations, and the HBV-DNA became undetectable [9]. A further patient with chronic HBV and cryoglobulinemic vasculitis who received entecavir obtained an improvement of the vasculitis and a suppression of the HBV-DNA. However, after more than 5 years after the therapy, the patient died due to a cerebral diffuse large B-cell lymphoma [9].

The reactivation of the hepatitis B virus following chemotherapy is a significant issue. Increased risks of liver-related morbidity and mortality due to an HBV reactivation have been documented in a number of previous research studies. The use of prophylactic lamivudine considerably reduced the frequency of HBV reactivation and chemotherapy discontinuation, with a trend towards an increased overall survival, as shown by a number of other investigations [76].

## 5. Conclusions

Chronic HBV infections can lead to rheumatic, nephrologic, and hematologic manifestations, and they can impact on patient mortality.

NAs treatment is expected to improve the mildest extra-hepatic symptoms, but immune modulating and immune suppressive agents (such as high dose IgG, rituximab high dose corticosteroids, and plasmapheresis) are required to handle the major renal, neurologic and hematologic manifestations.

Apart from combining the antiviral medications with a target therapy for extra-hepatic diseases, there are currently no specific recommendations that are available for the management of the patients with HBV-related disorders.

It is worth underlining that the inactive carriers of HBsAg that are given corticosteroids or immunosuppressive drugs present with an increased probability of viral reactivation. For this reason, an antiviral regimen against HBV is mandatory to avoid the HBV reactivation or its recurrence with the possible rapid progression of a liver disease. Therefore, starting 1–2 weeks before this occurs, immunosuppressants should be considered in these cases.

Further investigations on large populations of patients with HBV-related extraepatic manifestations will hopefully allow us to design the optimal therapy managements.

## Figures and Tables

**Table 1:** Extrahepatic manifestations of chronic hepatitis B.

Reported Condition
Mixed cryoglobulinemia vasculitis
Serum sickness-like syndrome
Non-rheumatoid arthritis
Rheumatoid arthritis
Panarteritis nodosa
Glomerulopathies
Non-Hodgkin Lymphoma

**Table 2:** Nucleotide anologues (NAs) therapy in patients with HBV-related cryoglobulinemic vasculitis.

Study, Year	Patients N	Antiviral Agent, Dose = Duration, Weeks (w), Month (m)	Other Treatment	Clinical Manifestation (N)	Cryoglobulinemic Vasculitis Response
Boglione et al. [5]	7	Telbivudine 600 mg/day: 7 (100%) = 48 w		Purpura: 3 (43) Peripheral neuropathy: 4 (57) Skin ulcer: 2 (29) Chronic hepatitis: 7 (100)	**CR**: Purpura: 3 (43) Peripheral neuropathy: 2 (50) **NR**: Peripheral neuropathy: 2 (50) Skin ulcer: 2 (100)
Terrier et al. [6]	3	Lamivudine 100 mg/day: 1 (33) Entecavir 0.5 mg/day: 2 (67)	PE + CS + RTX: 1 (33) PE + CYC+ CS + RTX: 1 (33)	Purpura: 2 (67) Arthralgias: 2 (67) Glomerulonephritis: 3 (100) Chronic hepatitis: 3 (100)	**CR**: Purpura: 2 (100) Arthralgias: 2 (100) Glomerulonephritis: 1 (1) Glomerulonephritis (2)
Mazzaro et al. [7]	7	Entecavir (5) = 48 m Adefovir (1) = 48 m Lamivudine (1) = 48 m	CS alone (1)	Type II (7) Purpura (7) Arthralgias (7) Ulcer on the leg (1) Chronic hepatitis (6) Cirrhosis (1)	**CR**: Purpura (7) Arthralgias (5) Ulcer on the leg (1) **NR**: Arthralgias (2)
Li et al. [8]	9	Entecavir (7) = 16 m Lamivudine (2) = 12 m	CS alone (3) CS + CYC(1) CS + PE + RTX (1) CS + PS + MMF (1) R (9)	Purpura (4) Arthralgias (2) Per neuropathy (2) Gastrointestinal Vasculitis (2) Glomerulonephritis (9)	**CR**: Glomerulonephritis (2) Purpura (2), Arthralgias (2), Per neuropathy (2) **PR**: Glomerulonephrirtis (3) **NR**: Glomerulonephritis (4) (dialysis in 4 patients, 2 died)
Mazzaro et al. [9]	18	Entecavir: 11(78%) = 66 m Tenofovir: 6 (67%) = 67 m Lamivudine: 1 (5%) = 59 m	Peg-IFN alone: 3 (38%) CS associated Nas: 4 (22%) PE associated NAs: 4 (22%) RTX associated NAs: 2 (11%)	Purpura: 18 (100%) Arthralgias: 11 (61%) Ulcer on the leg: 3 (17%) Sjogren S.: 5 (28%) Peripheral neuropathy: 11 (61%) Chronic hepatitis: 4 (22%) Cirrhosis: 1 (6%) Glomerulonephritis: 1 (6%) NHL: 2 (11%)	**RC**: Purpura: 14 (78%) Arthralgias: 8 (73%) Ulcex: 2 (67%) Sjogren S.: 2 (40%) Peripheral neuropathy: 6 (55%) **NR**: Purpura: 2 (11%) Arthralgias: 3 (27%) Neuropathy: 5 (45%) Glomerulonephritis: 1 (100%) NHL: 2 (100%)

GN: glomerulonephritis, CS: Corticosteroid, CYC: cyclophosphamide, RTX: Rituximab, MMF: mycophenolate, PE: plasma exchange. CR: complete response, PR: partial response, NR: non responder.
